# FLIP-based autophagy-detecting technique reveals closed autophagic compartments

**DOI:** 10.1038/s41598-022-26430-5

**Published:** 2022-12-27

**Authors:** Hajime Tajima Sakurai, Satoko Arakawa, Saori Noguchi, Shigeomi Shimizu

**Affiliations:** Department of Pathological Cell Biology, Medical Research Institute, Tokyo Medical and Dental University (TMDU), 1-5-45 Yushima, Bunkyo-Ku, Tokyo, 113-8510 Japan

**Keywords:** Biological techniques, Cell biology

## Abstract

Autophagy results in the degradation of cytosolic components via two major membrane deformations. First, the isolation membrane sequesters components from the cytosol and forms autophagosomes, by which open structures become closed compartments. Second, the outer membrane of the autophagosomes fuses with lysosomes to degrade the inner membrane and its contents. The efficiency of the latter degradation process, namely autophagic flux, can be easily evaluated using lysosomal inhibitors, whereas the dynamics of the former process is difficult to analyze because of the challenges in identifying closed compartments of autophagy (autophagosomes and autolysosomes). To resolve this problem, we here developed a method to detect closed autophagic compartments by applying the FLIP technique, and named it FLIP-based Autophagy Detection (FLAD). This technique visualizes closed autophagic compartments and enables differentiation of open autophagic structures and closed autophagic compartments in live cells. In addition, FLAD analysis detects not only starvation-induced canonical autophagy but also genotoxic stress-induced alternative autophagy. By the combinational use of FLAD and LC3, we were able to distinguish the structures of canonical autophagy from those of alternative autophagy in a single cell.

## Introduction

Macroautophagy (hereafter, autophagy) is a cellular process that engulfs various cytosolic components with double-layered membranes called phagophores, and degrade them to recycle their ingredients. Autophagy proceeds in steps involving structures with characteristic morphologies; i.e., phagophore elongation, phagophore bending, and self-fusion to enclose cytosolic components, forming autophagosomes. Autophagosomes then fuse with lysosomes to become autolysosomes, in which sequestered components are degraded^1,2^. In the process, the generation of autophagosomes and autolysosomes to isolate cargos and degrade them, respectively, are characteristic and important steps of autophagy. The efficiency of the latter step can be measured by inhibiting protein degradation using bafilomycin A1 (BafA1), chloroquine, and E64d plus pepstatin^1,3^. In contrast, no versatile method for the detection autophagosomes has been established yet, despite several methods being proposed. One appropriate method is electron microscopy (EM). In 2-dimensional (2D) EM, double-membrane structures enclosing cargos are candidates for autophagosomes. Phagophores (open structures) and autophagosomes (a proportion of the closed compartments) can be roughly judged by their membrane features and by the properties of the enclosed contents^4–7^. In addition, 3D-EM is a useful method to directly distinguish phagophores and autophagosomes, but it requires a special EM machine^4,8,9^. As an easier method, spatiotemporal analysis of syntaxin 17 (Stx17) has been proposed, which is based on its accumulation just on the phagophore-closing site^5^. Cell-membrane permeabilization followed by GAPDH immunostaining has also been proposed. This procedure enables the detection of autophagosomes/autolysosomes because cytosolic free GAPDH, but not GAPDH enclosed in autophagic vacuoles, is washed out from the cells^10^. Two-step immunostaining of LC3 has also been reported; cytosolic LC3 is first immunolabelled after weak permeabilization of the cell membrane without any fixation, and then cells are normally immunostained using a different anti-LC3 antibody. The membrane-enclosed LC3 is not detected by the first immunolabelling, and hence closed autophagosomes/autolysosomes containing LC3 can be recognized only by the second anti-LC3 antibody^11^. The principle of this procedure is reasonable, and the methods are sometimes useful to detect autophagosome generation. However, it requires optimal membrane permeabilization followed by cell fixation, and hence the procedure is difficult. Therefore, a convenient and reliable procedure has not yet been developed to date. We therefore established a novel procedure to detect closed autophagic compartments using a photobleaching technique.

Recently, accumulating lines of evidence have shown the important roles of an alternative type of autophagy originating from the Golgi membrane, namely alterative autophagy or Golgi membrane-associated degradation (GOMED)^12–14^. Alternative autophagy proceeds with the generation of phagophores, autophagosomes, and autolysosomes, similarly to canonical autophagy, but using *trans-*Golgi membranes instead of ER and mitochondria-associated membranes. Molecularly, alternative autophagy involves ULK1 and BECN1/Beclin1, but not ATG5, ATG7, ATG9, LC3, and STX17/Syntaxin17^15–18^. Canonical and alterative autophagy are activated differently in a stimulus/context-dependent manner, and degrade different cargos. Unlike canonical autophagy, methods to detect alternative autophagy have not been fully developed yet, and we hence tested our photobleaching technique for this purpose.

## Results and discussion

### Photobleaching of cytosolic fluorescent proteins highlights autophagic contents

**Figure 1:**
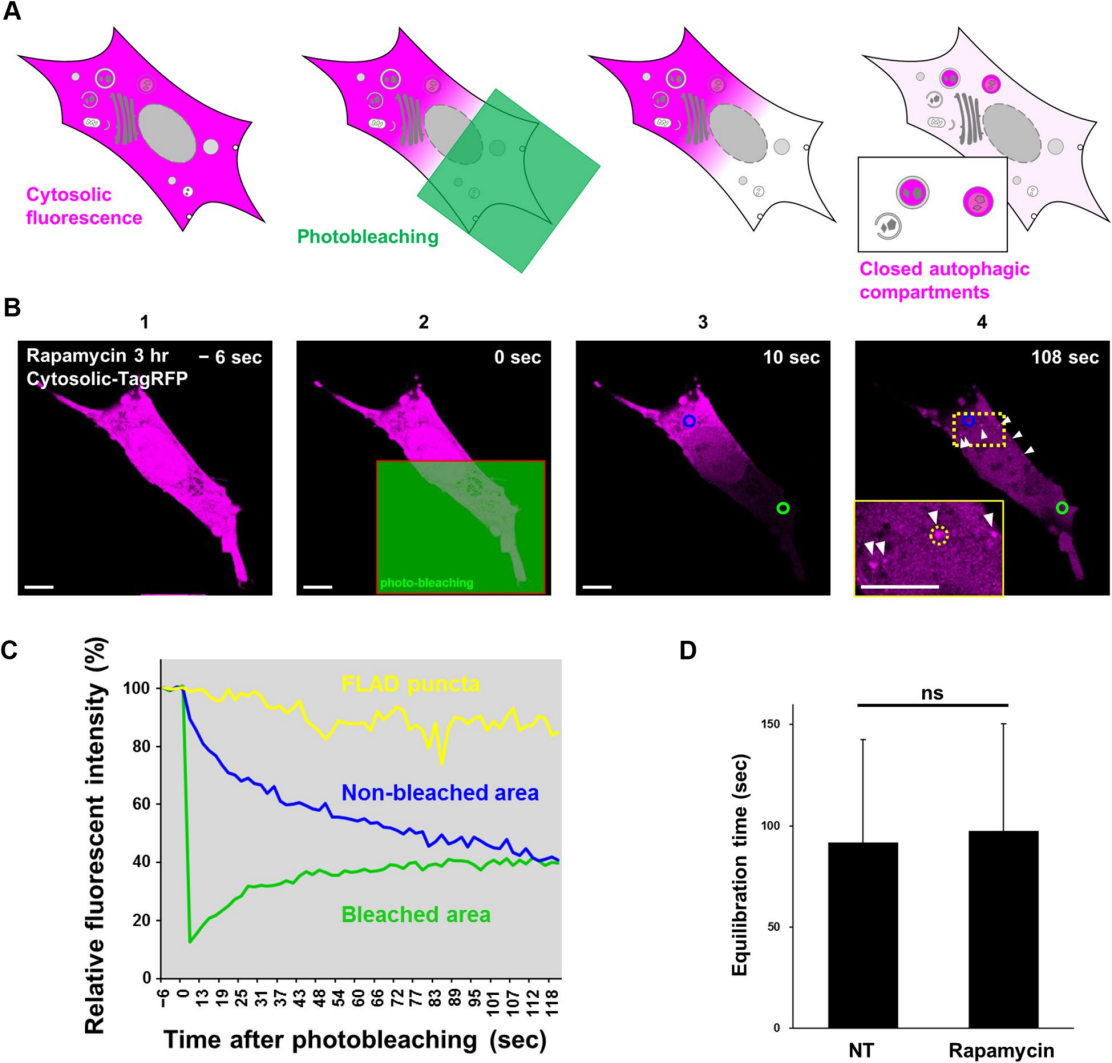
Establishment of the FLAD assay. **(A)** Schematic model of the FLAD assay. **(B)** Representative images of a cell subjected to the FLAD assay. Wild-type MEFs transiently expressing TagRFP were treated with 0.5 $\upmu$M rapamycin for 3 h to induce canonical autophagy. Then, the FLAD assay was performed. Image 1: just before photobleaching. Image 2: during photobleaching. Half of the cell area in the confocal plane (green rectangle) was photobleached. Image 3: just after photobleaching. Green and blue circle: region used to measure fluorescence intensity of the bleached area and non-bleached area, respectively. The TagRFP fluorescence disappeared only in the bleached area. Image 4: 108 s after photobleaching. Several FLAD^+^ puncta were observed as bright TagRFP puncta (arrowheads) in the non-bleached area. The ROI is shown by the yellow dotted rectangle and the magnified image is shown in the inset. Bars = 10 $\upmu$m. **(C)** Time-dependent alterations in fluorescence intensity in the bleached area (green circle in image 3 of B), the non-bleached area (blue circle in image 3 of B), and FLAD^+^ puncta (yellow dotted circle in image 4 of B) was monitored continuously. Note that the blue and green circles are almost an equal distance from the borderline of the bleached area. **(D)** Equilibration time of wild-type MEFs treated with or without rapamycin (0.5 µM). Cytoplasmic intensities were equilibrated at 91.1 ± 50.6 s in control cells (n = 5) and 97.4 ± 53.1 s in cells treated with rapamycin for 3 h (n = 4). Error bars indicate the S.D. Statistical analysis was performed by the Student *t*-test. ns: no significant difference.

The generation of closed compartments is a unique characteristic of autophagy, and its quantification is important to analyze autophagy dynamics. To develop a method for the live-imaging of closed autophagic compartments, we decided to apply the photobleaching technique, which is conventionally used for Fluorescence Loss In Photobleaching (FLIP) and Fluorescence Recovery After Photobleaching (FRAP)^19,20^. Fluorescent tag proteins, such as GFP, are vastly used in the analyses of subcellular localization, based on the idea that the fluorescent proteins themselves are neutrally cytosolic dispersed localization^21^. When about half of the cell area is photobleached, the fluorescence signal in the cytosol is transiently reduced, followed by gradual recovery owing to influx from the non-bleached area (Fig. 1A). In the non-bleached area, the cytosolic fluorescence signal gradually decreases because of fluorescent protein efflux into the bleached area by cytoplasmic streaming. If there are closed autophagic compartments (autophagosomes and autolysosomes) within the non-bleached area, the fluorescent proteins incorporated into these structures are evaded from streaming, so that they are detectable as fluorescent puncta (Fig. 1A). The phagophores (open structures) should not be detected as fluorescent puncta. We named this procedure as the FLIP-based Autophagy Detecting (FLAD) technique.

For validation of this technique, we first performed the FLAD assay using TagRFP-expressing mouse embryonic fibroblasts (MEF). MEFs were sufficiently photobleached in about half the area of the cell within 10 s (see details in Methods, Suppl. Movie 1). The fluorescence intensity in the bleached area (green rectangle) was rapidly decreased, and then gradually increased after photobleaching (Fig. 1B,C). In contrast, the fluorescence intensity in the non-bleached area was gradually decreased and reached an equivalent level as that in the bleached area at approximately 115 s in this experiment (Fig. 1C). Expectedly, we observed several TagRFP puncta in the non-bleached area (Fig. 1B, arrowheads; and 1C, yellow line), indicating the existence of closed compartments containing fluorescent proteins. By multiple experiments, the protein distribution time (the time for the fluorescence intensity to become equal in bleached and unbleached areas) was about 100 s, regardless of treatment with rapamycin, which induces autophagy via mTor inhibition (Fig. 1D).

**Figure 2:**
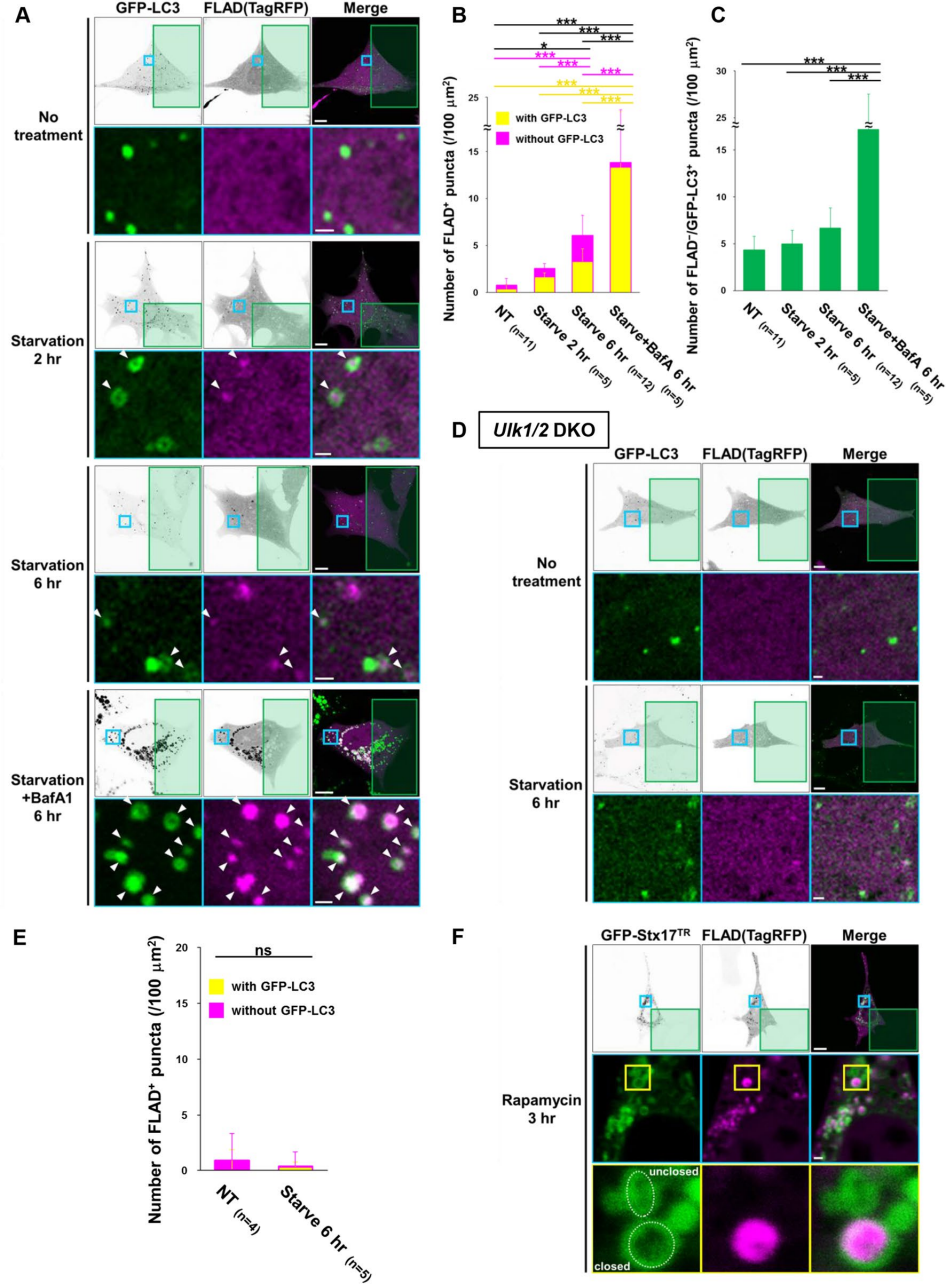
FLAD assay for the detection of canonical autophagy. **(A-C)** Wild-type MEFs stably expressing GFP-LC3 were transiently coexpressed with TagRFP. After 24 h, cells were starved in the presence or absence of 10 nM BafA1. Then, cells were photobleached in the green areas for the indicated times, and fluorescence images were acquired at least 3 min later (hereafter described as “equalization”). In **(A)**, representative images are shown. Whole-cell single-color images are displayed as black/white images to make the puncta easier to see. ROIs are indicated as blue squares, and their magnified images are shown in the lower panels. Bars = 10 $\upmu$m and 1 $\upmu$m in the upper and lower panels, respectively. In **(B)**, the number of FLAD^+^ puncta with GFP-LC3 (yellow) and without GFP-LC3 (magenta) in the non-bleached area was quantified. In **(C)**, the number of GFP-LC3 single puncta was quantified. **(D, E)** ULK1/2-deficient MEFs transiently expressing GFP-LC3 and TagRFP were starved for 6 h, and photobleached (green rectangles). Then, cell images were acquired after equalization. In **(D)**, whole-cell fluorescence images are displayed as black/white images. ROIs are indicated by the blue squares, and their magnified images are shown in the lower panels. Bars = 10 $\upmu$m and 1 $\upmu$m in the upper and lower panels, respectively. In **(E)**, the number of FLAD^+^ puncta with GFP-LC3 (yellow) and without GFP-LC3 (magenta) in the non-bleached area was quantified. **(F)** Wild-type MEFs stably expressing GFP-Stx17^TR^ were transiently expressed with TagRFP, and photobleached in the green area at 3 h after rapamycin treatment (0.5 µM). After equalization, fluorescence images were acquired. ROIs are indicated as blue and yellow squares, and their magnified images are shown in the middle and lower panels, respectively. Bars = 10 $\upmu$m and 1 $\upmu$m in the upper and middle panels, respectively. In the bottom panels, the dotted lines indicate representative structures of regular spherical GFP-Stx17 (closed compartment) and ellipsoidal GFP-Stx17 (open structure). Note that only regular spherical GFP-Stx17 contained FLAD signals. In (B, C, E), the total number of images used for analysis is given as the *n*. Data are shown as the mean ± S.D. *p < 0.05, ***p < 0.001, ns, no significant difference (one-way ANOVA followed by the Tukey post hoc test and Student *t*-test).

We next investigated whether FLAD^+^ puncta indicate autophagic vacuoles. For this purpose, we expressed TagRFP together with GFP-LC3, which is a widely used marker of autophagic vacuoles^22^, into MEFs. In untreated cells, FLAD^+^ puncta were rarely observed. However, upon starvation, FLAD^+^ puncta were observed and increased time-dependently, and many of them colocalized with GFP-LC3^+^ puncta (Fig. 2A,B, and Suppl. Fig. 1), suggesting that FLAD^+^ puncta are autophagic vacuoles. Although there were a considerable number of FLAD^+^ puncta lacking GFP-LC3 signals, particularly in the cells starved for 6 h (Fig. 2A), these puncta are thought to be autolysosomes in which GFP-LC3 signals have disappeared because of the low pH and protein degradation within the autolysosomes (GFP is more sensitive to acidic conditions and protein degradation than TagRFP)^23,24^. This was confirmed by the almost complete merging of FLAD^+^ puncta and GFP-LC3^+^ puncta by the addition of BafA1, which inhibits GFP-LC3 disappearance by suppressing lysosomal acidification and autolysosomal degradation (Fig. 2A,B). Although the possibility is low, some of FLAD^+^/GFP-LC3^−^ puncta may also be structures involved in alternative autophagy (described later). The FLAD^-^/GFP-LC3^+^ puncta (open GFP-LC3^+^ structures) are thought to be phagophores, and their number was constant regardless of starvation (Fig. 2A,C). Consistent results were obtained when cells were treated with rapamycin (Suppl. Fig. 2).

To validate the FLAD assay, we compared the results of the FLAD assay with results of the modified Cell-membrane permeabilization assay, which is a previously reported assay for the detection of closed autophagic compartments^10^. For the latter assay, we expressed TurboGFP, induced autophagy by starvation, and permeabilized and added trypsin to the cells, by which we were able to detect closed autophagic compartments, because cytosolic free TurboGFP, but not TurboGFP enclosed in autophagic vacuoles, are degraded^10^. When we counted the number of TurboGFP puncta before and after the induction of starvation, the numbers were comparable with the FLAD^+^ puncta (Fig. 2B and Suppl. Fig. 3A), validating the correctness of the FLAD assay. We also tried using a combination of the Cell-membrane permeabilization assay and the FLAD assay. As shown in the representative images, red FLAD puncta colocalized at a high frequency with TurboGFP puncta from the Cell-membrane permeabilization assay (Suppl. Fig. 3B). These two findings validate the correctness of the FLAD assay.

The successful detection of closed autophagic compartments by FLAD was further confirmed when MEFs lacking ULK1/2 and ATG5 were starved. These molecules are essential for canonical autophagy, and hence FLAD^+^ puncta were not generated upon starvation (Fig. 2D,E, and Suppl. Fig. 4). Although we were concerned that FLAD detects not only autophagosomes but also other organelles, FLAD signals were not merged with major organelles, such as mitochondria and the ER, even after rapamycin treatment (Suppl. Fig. 5). The difference between wild-type MEFs and ATG5-deficient or ULK1/2-deficient MEFs supported that FLAD specifically detects autophagy-dependent structures.

A recent study reported that Stx17 is recruited on phagophores immediately before the closure event. Because closed compartments and open structures form regular spherical bodies and ellipsoidal bodies, respectively, the oblateness of GFP-Stx17^+^ structures have the potential to be an index of membrane closure^5^. Interestingly, the FLAD assay using MEFs expressing GFP-Stx17 demonstrated that FLAD was detected in the regular spherical GFP-Stx17^+^ structures but not ellipsoidal structures (Fig. 2F), indicating that the FLAD assay enables live-imaging of closed autophagic compartments of canonical autophagy.

### FLAD assay provides a novel approach to detect Atg5-independent autophagy

**Figure 3:**
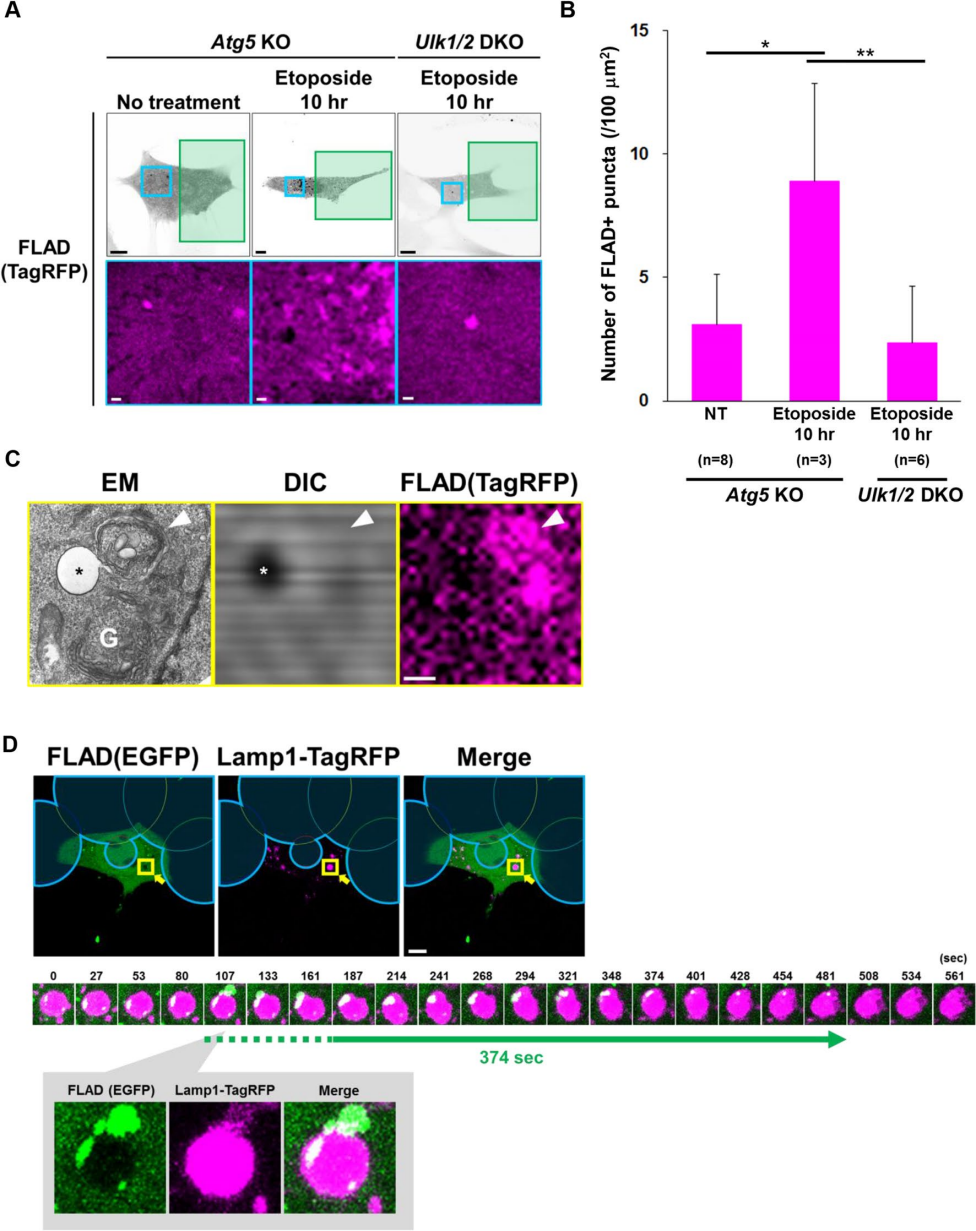
FLAD assay for the detection of alternative autophagy. **(A, B)** TagRFP was transiently expressed both in Atg5- and Ulk1/2-deficient MEFs. The cells were treated with or without 10 $\upmu$M etoposide for 10 h to induce alternative autophagy, and then photobleached in the green area. In **(A)**, representative images are shown. Bars = 10 $\upmu$m. ROIs are indicated as blue rectangles, and magnified images are shown in the lower panels. Bars = 1 $\upmu$m. In **(B)**, the number of FLAD^+^ puncta in the non-bleached area was quantified. The total number of images used for analysis is given as the *n*. Data are shown as the mean ± S.D. *p < 0.05, **p < 0.01 (one-way ANOVA followed by the Tukey post hoc test). **(C)** Identification of FLAD^+^ puncta as double-layered structures by CLEM analysis. ATG5-deficient MEFs expressing TagRFP were treated with etoposide (10 µM) for 10 h. After equalization following photo-bleaching, cells were fixed with 0.75% paraformaldehyde/1.5% glutaraldehyde, and their images were observed by fluorescence microscopy. Cells were subsequently fixed with 1% OsO_4_ and observed by EM. Bar = 10 $\upmu$m. Adjustment between fluorescence microscopy and EM images, and lower magnification images of the ROI are indicated in Fig. S5. The representative FLAD^+^ punctum indicated by the arrowhead in the right panel corresponds to the double-membrane autophagic structure in the left EM panel. “G” indicates the golgi apparatus, where alternative autophagic membranes originate. **(D)** ATG5-deficient MEFs expressing Lamp1-TagRFP were transiently expressed with EGFP, treated with etoposide (10 µM) for 10 h, and then photobleached with a 488 nm laser in the area of the blue circles in the upper panels. ROIs are indicated by yellow squares, and the magnified time-lapse images are shown in the middle panels. In the lower panels, magnified and separated single-color images at 107 s are presented. Note that the FLAD signal composed of EGFP was fused with an autolysosome at 107 s, and then the signal disappeared after 374 s. Bars = 10 $\upmu$m.

We next analyzed whether the FLAD assay is applicable not only to Atg5-dependent canonical autophagy, but also to Atg5-independent alternative autophagy. For this aim, ATG5-deficient MEFs were treated with etoposide, which induces alternative autophagy via genotoxic stress^18^. Expectedly, we found a substantial increase in FLAD^+^ puncta upon etoposide treatment in ATG5-deficient MEFs (Fig. 3A,B). In contrast, FLAD^+^ puncta were not generated in ULK1/2-deficient cells, which lack both types of autophagy (Fig. 3A,B). Unlike rapamycin-treated wild-type MEFs, the colocalization of FLAD^+^ puncta and GFP-Stx17^+^ puncta was rare in etoposide-treated ATG5-deficient MEFs, supporting that these FLAD^+^ puncta are structures of alternative autophagy (Suppl. Fig. 6). To confirm whether the FLAD^+^ puncta in etoposide-treated ATG5-deficient MEFs correspond to closed alternative autophagic compartments, we performed two additional experiments. Correlative light and electron microscopy (CLEM) is a method to identify the structures of fluorescent signals, and we found that the FLAD^+^ puncta corresponded to autophagosomes detected on EM (Fig. 3C and Suppl. Fig. 7). We further performed the FLAD assay using EGFP instead of TagRFP, and analyzed the colocalization of FLAD^+^ puncta with Lamp1-TagRFP, a marker of lysosomes and autolysosomes. Because EGFP signals gradually disappear within autolysosomes, it is useful for measuring autophagic degradation dynamics. As expected, we observed that the green FLAD signals merged well with Lamp1-positve signals upon etoposide treatment, and green signals disappeared gradually, displaying the dynamics of FLAD^+^ puncta. Interestingly, EGFP degradation in an autolysosome of alternative autophagy only took 374 s, which was approximately equivalent to that in canonical autophagy (~ 420 s) (Fig. 3D). Although it has been demonstrated that EGFP is degraded slowly (> 30 min) by canonical autophagy in rapamycin-treated ATG5-deficient MEFs^5^, the time required for the degradation was clearly shorter in alternative autophagy, implying that alternative autophagy is different from slowly progressing canonical autophagy in cells lacking ATG5.

### Simultaneous analysis of canonical autophagy and alternative autophagy using the FLAD assay

**Figure 4:**
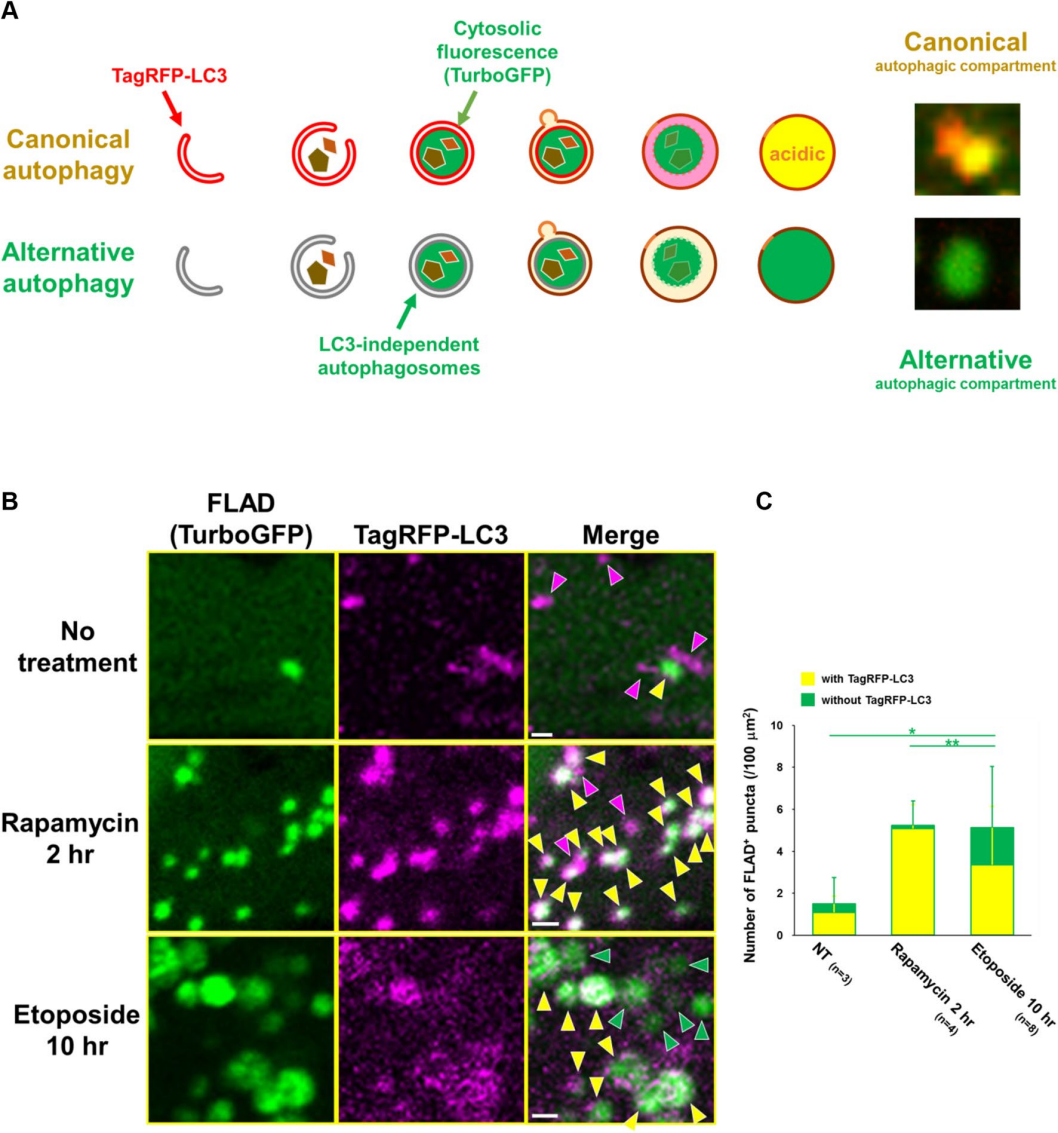
Simultaneous detection of canonical and alternative autophagy using FLAD and TagRFP-LC3. **(A)** Schematic model of the assay. Closed compartments from canonical autophagy can be detected as yellow puncta because of the signals of TagRFP-LC3 (red) and FLAD (green), whereas those from alternative autophagy can be detected as green puncta because of the signal of FLAD (green). **(B, C)** Wild-type MEFs transiently coexpressing TagRFP-LC3 and TurboGFP were treated with etoposide (10 $\upmu$M) for 10 h or rapamycin (0.5 $\upmu$M) for 2 h, and then the FLAD assay was performed. In (**B**), representative images are shown. Green puncta and white puncta indicate closed compartments from alternative autophagy and canonical autophagy, respectively. Magenta puncta indicate open structures (phagophores) from canonical autophagy. In **(C)**, the number of FLAD^+^ puncta with tagRFP-LC3 (yellow) and without tagRFP-LC3 (green) in the non-bleached area was quantified. The total number of images used for analysis is given as the *n*. Data are shown as the mean ± S.D. *p < 0.05, **p < 0.01 (one-way ANOVA followed by the Tukey post hoc test).

Both canonical autophagy and alternative autophagy occur in the same cell, and the proportion is context dependent; i.e., type of stimulus, cell, and circumstance. Elucidating their ratio is important to understand what is happening in the cell, and it is possible to perform the FLAD assay using TurboGFP together with TagRFP-fused *trans*-Golgi protein, because they are both resistant to lysosomal acidification and degradation^24,25^ and only alternative autophagy originates from the *trans*-Golgi membrane (Suppl. Fig. 8). It is also possible to use TagRFP-LC3, instead of TagRFP-fused *trans*-Golgi protein. Because the FLAD assay recognizes both types of autophagy, and TagRFP-LC3 labels only canonical autophagy, TagRFP-LC3^+^/TurboGFP^+^ (yellow) structures and TagRFP-LC3^−^/TurboGFP^+^ (green) structures indicate closed canonical autophagic compartments and closed alternative autophagic compartments, respectively (Fig. 4A). We applied this procedure for analyzing autophagy induced by etoposide and rapamycin. Upon rapamycin treatment, the number of FLAD^+^ puncta reached approximately 5.24 puncta in 100 $\upmu$m^2^, and most of them were yellow puncta derived from canonical autophagy (Fig. 4B,C, and Suppl. Fig. 9). In contrast, in etoposide-treated MEFs, the number of FLAD^+^ puncta also reached about 5.13 puncta in 100 $\upmu$m^2^, and about 65% of them were yellow puncta and the remaining puncta were green (Fig. 4B,C, and Suppl. Fig. 9), indicating that etoposide induced both canonical and alternative autophagy within one cell. These results are consistent with previous studies, in which rapamycin specifically induces canonical autophagy, whereas etoposide induces both types of autophagy^18,26^. Taken together, our results demonstrate that the FLAD assay is useful for the detection of closed autophagic compartments. Furthermore, both canonical and alternative autophagy can be distinguished within one cell by the combinational use of the FLAD assay and LC3.

## Materials and methods

### Reagents and instruments

Rapamycin and BafA1 were obtained from Santa Cruz Biotechnology and Focus Biomolecules, respectively. MitoTracker Green FM was purchased from Thermo Fisher Scientific. Etoposide was purchased from Sigma-Aldrich. Other chemicals, including CCCP, were purchased from Nacalai Tesque. Fluorescence images were obtained using LSM710 confocal laser-scanning microscopes (Zeiss). For CLEM observation, a JEM-1400Plus transmission electron microscope (JEOL) was used with a CCD camera (EM-14830RUBY2; JEOL).

### Cell culture and transfection

Wild-type and Atg5-deficient MEFs were harvested from mouse embryos on embryonic day 13.5, and immortalized using the SV40 T antigen^27^. MEFs were cultured in high-glucose Dulbecco’s modified Eagle’s medium (Nacalai) supplemented with 2 mM L-glutamine, 1 mM sodium pyruvate, 0.1 mM nonessential amino acids, 10 mM HEPES/Na^+^ (pH 7.4), 0.05 mM 2-mercaptoethanol, 100 U/mL penicillin, 100 µg/mL streptomycin, and 10% (v/v) fetal bovine serum in a humidified 5% or 10% CO_2_ incubator at 37 °C. For the analysis of starvation-induced autophagy, cells were cultured in Hanks’ Balanced Salt Solution (HBSS( +), Nacalai) after washing with HBSS( +) three times.

For transient plasmid expression, MEFs (1 × 10^6^) were transfected with 1 μg plasmid DNA using the Neon transfection system (Thermo) or AMAXA Cell Line Nucleofector Kit V (Lonza) according to the supplier’s protocol (1,300 V, 20 ms, two times, or program U-20). The expression plasmids *GFP-LC3* and *LAMP1-TagRFP* were modified from the plasmids *TagRFP-LC3* and *LAMP1-TagRFP*, respectively, which were used in a previous study^28^. The *GFP-STX17*^*TR*^-expression plasmid was made by truncating the *GFP-STX17*^*WT*^ plasmid^5^. We also used mCherry-tagged Beta-1,4-galactosyltransferase1 expressing plasmid^29^. The transfection efficiency was more than 75%, as assessed by the GFP fluorescence of the cells.

### FLAD assay

In each assay, MEFs were transiently transfected with localization-free fluorescence-expressing plasmid DNA. The expression plasmids *pEGFP-C1* (lysosomal degradable GFP), *pMaxGFP* (acid-proof GFP) and *pTagRFP-C* (acid-proof RFP) were purchased from Addgene, Lonza, and Evrogen, respectively. The transfection efficiency was more than 75%, as assessed by the fluorescence of the cells. A few days after transfection, cells with moderate expression (without any aberrant aggregations; see first panel of Fig. 1B) were selected and fluorescence images before photobleaching were obtained using an LSM710 confocal laser-scanning microscope (Zeiss). Almost half of the total cell area was set as the region of interest (ROI) for photobleaching (excitation output level: 100%; iteration: 10–50) using the ZEN application (Zeiss). Subsequently, fluorescence images were obtained after adequate intervals for cytosolic background-equilibration (about 2 min). Note that brighter cytosolic fluorescent-protein expression and sufficient photobleaching enables the acquisition of images with better signal/noise contrast.

### Cell-membrane permeabilization assay

The cell-membrane permeabilization assay was performed according to the method reported previously^10^, with some modifications. Briefly, WT MEFs were transiently transfected with the *pMaxGFP* plasmid, as described above. After 24 h, cells were permeabilized with 20 $\upmu$g/mL digitonin (Wako) for 1 min at room temperature, and immediately fixed with 4% PFA for 10 min at room temperature. The cells were then treated with 2.5 g/L trypsin (Nacalai) to degrade cytosolic TurboGFP for 2 min at 37 °C, and trypsin treatment was discontinued by the addition of DMEM with 10% FBS. Undegraded TurboGFP within autophagic compartments was detected using LSM710 confocal laser-scanning microscope (Zeiss).

### CLEM

MEFs cultured on glass-bottom dishes with grids were treated with etoposide to induce alternative autophagy, and were photobleached by the above-mentioned procedure. In equilibration time, samples were fixed with paraformaldehyde (0.75%)/glutaraldehyde (1.5%) in 0.1 M phosphate buffer (PB) at pH 7.4 at room temperature, and FLAD signals were observed immediately using an LSM710 fluorescence microscope. Subsequently, the samples were put on ice for 30 min. Thereafter, the samples were fixed with 2% glutaraldehyde in 0.1 M PB at 4 °C overnight. After fixation, the samples were washed three times with 0.1 M PB and postfixed with 1% OsO4 in 0.1 M PB at 4 °C for 1 h. After dehydration, ultrathin sections were stained with 2% uranyl acetate and lead stain solution (Sigma-Aldrich), and observed using a JEM-1400Plus electron microscope (JEOL) at 100 kV.

### Statistical analysis

All results are expressed as the mean ± standard deviation (S.D.). Statistical analyses were performed using Prism5 (GraphPad) software. Comparisons of multiple datasets were performed using one-way ANOVA followed by the Tukey post hoc test for multiple comparisons, or the two-tailed unpaired Student *t*-test. A *p*-value of less than 0.05 was considered to indicate a statistically significant difference between two groups.

## Supplementary Information

Supplementary Information 1.

Supplementary Information 2.

Supplementary Video 1.

## Data Availability

All data supporting the findings of this study are available from the corresponding author upon request.
