# Supplementary material for: FLIP-based autophagy-detecting technique reveals closed autophagic compartments
Source: Sci Rep. 2022 Dec 27;12:22452. doi: 10.1038/s41598-022-26430-5 (PMC9794774; doi:10.1038/s41598-022-26430-5)
Supplement: Supplementary file 1 — Supplementary Information 1. [file 41598_2022_26430_MOESM1_ESM.docx]

**Supplemental Figure 1. Magnified images of the cells in Figure 2A**

Wild-type MEFs stably expressing GFP-LC3 were transiently coexpressed with TagRFP. After 24 hr, cells were starved in the presence or absence of 10 nM BafA1. Then, cells were photobleached in the green area at the indicated times, and fluorescence images were acquired after equalization. Whole-cell single-color images are displayed as black/white images to make the puncta easier to see. ROIs are indicated by blue squares, and shown in the bottom panels of Fig. 2A. Bars = 10 μm

**Supplemental Figure 2. FLAD assay for the detection of rapamycin-induced canonical autophagy**

Wild-type MEFs stably expressing GFP-LC3 were transiently coexpressed with TagRFP. After 24 hr, cells were treated with 0.5 μM rapamycin in the presence or absence of 10 nM BafA1. Then, cells were photobleached in the green area for the indicated times, and fluorescence images were acquired after equalization. Representative images are shown. Whole-cell single-color images are displayed as black/white images to make the puncta easier to see. ROIs are indicated by blue squares, and their magnified images are shown in the lower panels. Bars = 10 μm and 1 μm in the upper and lower panels, respectively.

**Supplemental Figure 3. Cell-membrane permeabilization assay for the detection of closed autophagosomes**

**(A)** Wild-type MEFs were transiently transfected with the TurboGFP plasmid. After 24 hr, cells were treated with or without starvation for 6 hr. Then, cells were permeabilized using 20 μg/mL digitonin for 1 min, followed by PFA fixation and trypsin treatment. The number of TurboGFP puncta that were protected from trypsin degradation was quantified. The total number of images used for analysis is given as the *n*. Data are shown as the mean ± S.D. **p* < 0.05 (Student *t*-test). **(B)** Identification of FLAD^+^ puncta as closed autophagosomes by the cell-membrane permeabilization assay. Wild-type MEFs were transiently transfected with TurboGFP and TagRFP, and photobleached only TagRFP in the green area at 6 hr after rapamycin treatment (0.5 µM). After equalization following photobleaching, cells were permeabilized by 20 μg/mL digitonin for 1 min, followed by PFA fixation and trypsin treatment. Bar = 10 μm.

**Supplemental Figure 4. FLAD assay for the detection of ATG5-deficient cells upon starvation**

ATG5-deficient MEFs transiently expressing GFP-LC3 and TagRFP were starved for 6 hr, and photobleached (area in the green rectangles). Then, cell images were acquired after equalization. Whole-cell fluorescence images are displayed as black/white images. ROIs are indicated by blue rectangles and are shown in the lower panels. Bars = 10 μm and 1 μm in the upper and lower panels, respectively.

**Supplemental Figure 5. Colocalization analysis of FLAD^+^ puncta with other organelle markers**

**(A)** *GFP-Cb1* (cytochrome b1; ER marker) and *TagRFP* were transfected into wild-type MEFs. 24 hrs later, cells were treated with 0.5 μM rapamycin for 2 hr and photobleached in the green area. ROIs are shown as blue rectangles, and their magnified images are shown in the lower panels. Bars = 10 μm and 1 μm in the upper and lower panels, respectively. **(B)** Wild-type MEFs transiently expressing TagRFP were preincubated with 1 μM Mito Tracker Green, and then treated with 0.5 μM rapamycin or 20 μM CCCP for 2 hr. Cells were photobleached in the green rectangular area. ROIs were magnified in the lower panels. Bars = 10 μm and 1 μm in the upper and lower panels, respectively.

**Supplemental Figure 6. No colocalization of FLAD^+^ puncta with Stx17 in alternative autophagy**

ATG5-deficient MEFs expressing GFP-Stx17^TR^ were treated with or without 10 μM etoposide for 10 hr to induce alternative autophagy, and then photobleached in the green area. After equalization, fluorescence images were acquired. Bars = 10 μm. ROIs are indicated as blue squares, and their magnified images are shown in the lower panels. Bars = 1 μm. Note that GFP-Stx17^TR^ signals were not merged with FLAD signals.

**Supplemental Figure 7. Low-magnification images of the cells in Figure 3C**

ATG5-deficient MEFs were transfected with *pTagRFP*, and treated with 10 µM etoposide for 10 hr. The green circled areas were photobleached. DIC and EM images were adjusted using the position of the vacuoles (asterisks). The ROIs indicated by the blue squares are magnified in the lower panels, and the ROIs indicated by yellow squares are highly magnified in Figure 3C. Bar = 10 μm

**Supplemental Figure 8. Colocalization analysis of FLAD^+^ puncta with *trans*-Golgi marker**

ATG5-deficient MEFs expressing TurboGFP and GalT-TagRFP (*trans*-Golgi marker) were photobleached in the blue area without any treatment. After equalization, fluorescence images were acquired. Bars = 10 μm. ROIs are indicated as yellow squares, and their magnified images are shown in the lower panels. Bars = 1 μm.

**Supplemental Figure 9. Low-magnification images of the cell in Figure 4B**

Wild-type MEFs transiently coexpressing TagRFP-LC3 and TurboGFP were treated with 0.5 μM rapamycin for 2 hr or 10 μM etoposide for 10 hr. After treatment for the indicated times, the cells were photobleached in the blue area. Whole-cell single-color images are displayed as black/white images to make the puncta easier to see. The ROIs are indicated by yellow squares, and their magnified images are shown in Figure 4B. Bars = 10 μm

**Supplemental Movie 1. Time-course display of TagRFP intensity distribution in Figure 1B**

Wild-type MEFs expressing TagRFP were treated with 0.5 μM rapamycin for 3 hr. TagRFP fluorescence in the cells was sequentially acquired and displayed as a heat map. First, high-fluorescence intensity (red) was detected throughout the cell (~0 sec). Next, photobleaching was performed in the red rectangular region at 0 sec, and a significant decrease in fluorescence (blue) was observed in the bleached area. Subsequent equalization recovered the fluorescence to a moderate intensity (green) in the bleached area. On the other hand, in the non-bleached area, the intensity of cytosolic fluorescence was found to be reduced to the same level, although there were some puncta (red, autophagic compartments) showing strong intensity (red). The TagRFP fluorescence intensity in the bleached area and non-bleached area (blue and green circles, respectively) was monitored and displayed as blue and green lines, respectively, in Figure 1C. Bars = 10 μm
