# Supplementary material for: FLIP-based autophagy-detecting technique reveals closed autophagic compartments
Source: Sci Rep. 2022 Dec 27;12:22452. doi: 10.1038/s41598-022-26430-5 (PMC9794774; doi:10.1038/s41598-022-26430-5)

Supplemental Figure 1

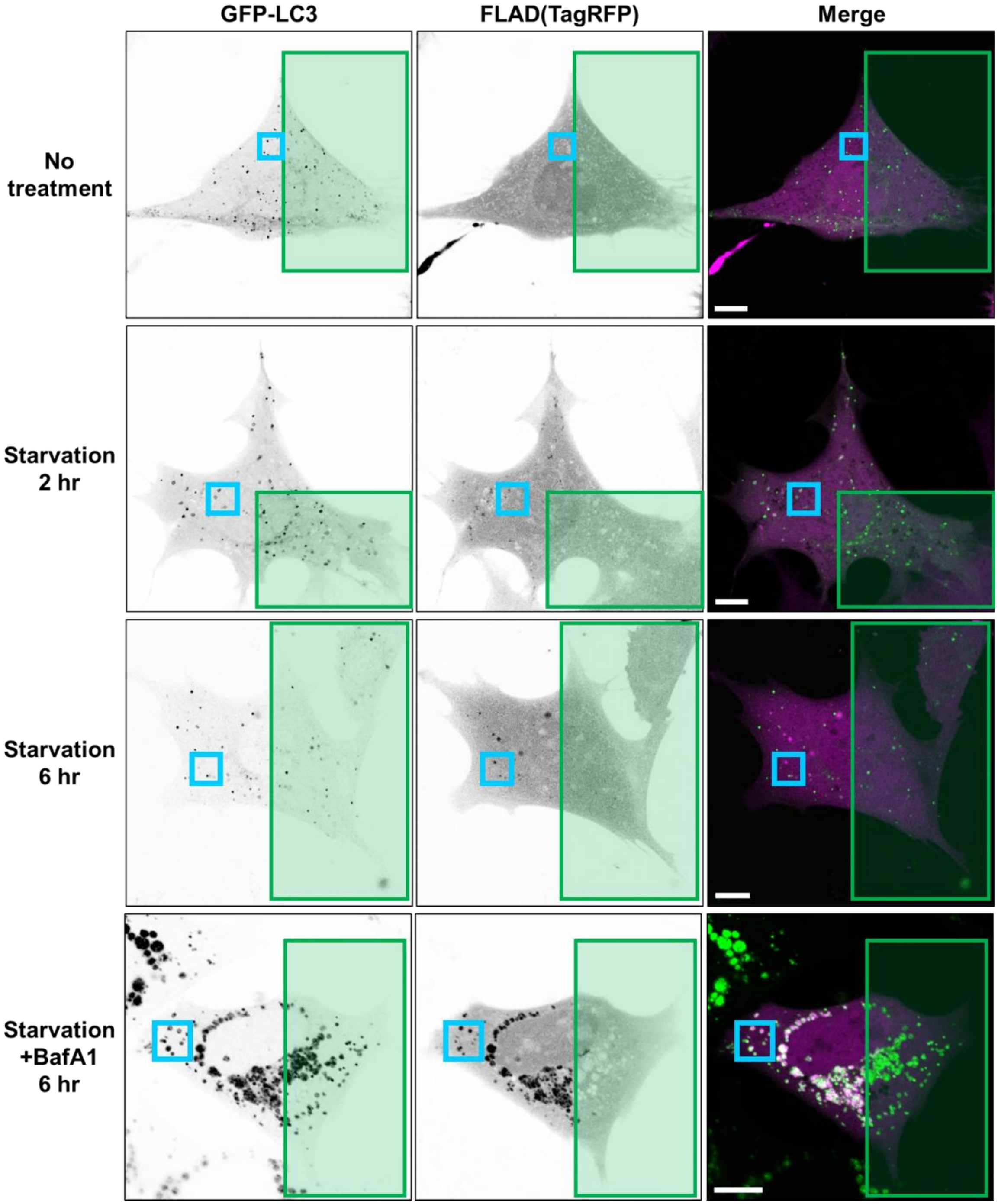

Supplemental Figure 2

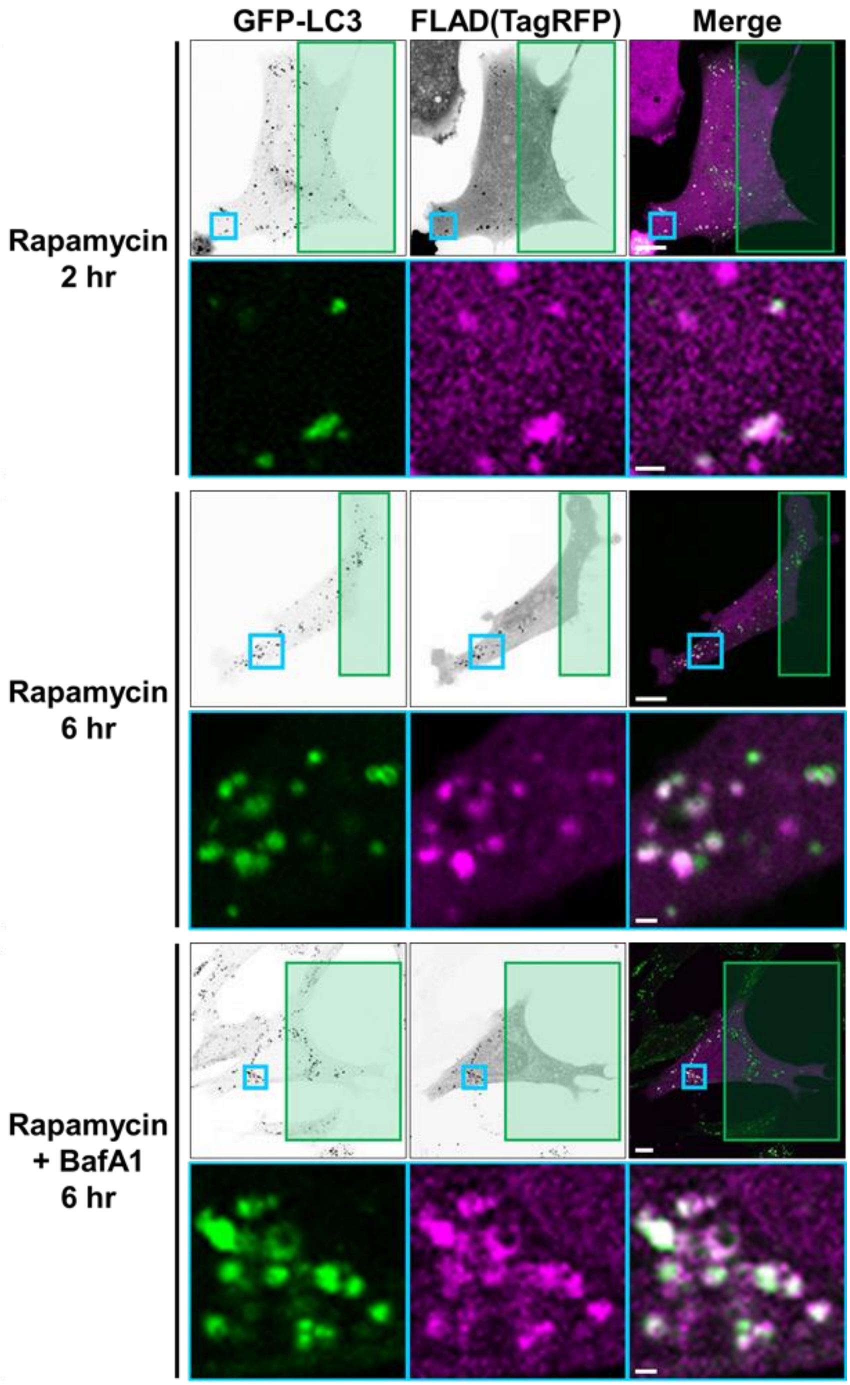

Supplemental Figure 3

A

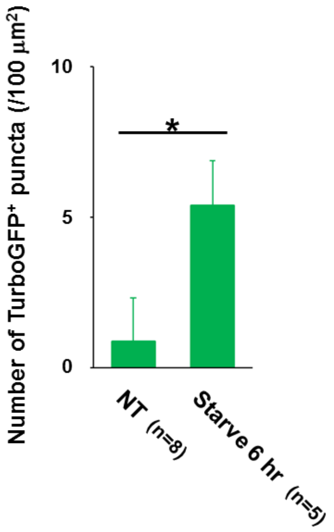

B

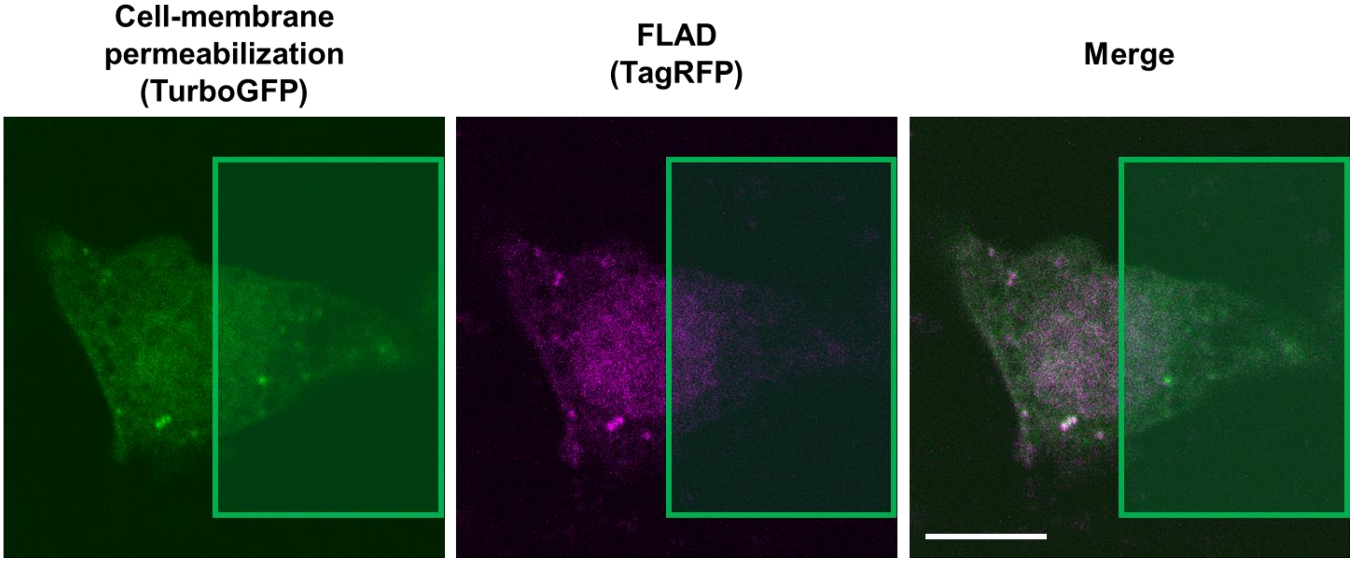

Supplemental Figure 4

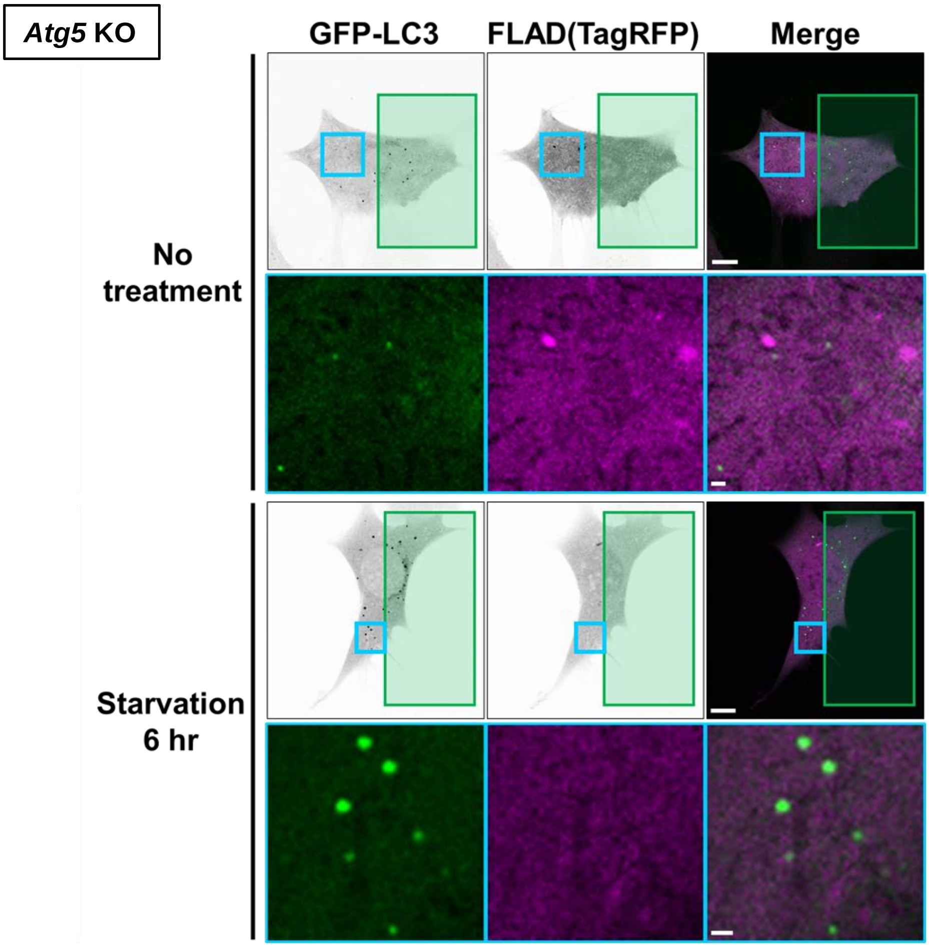

Supplemental Figure 5

A

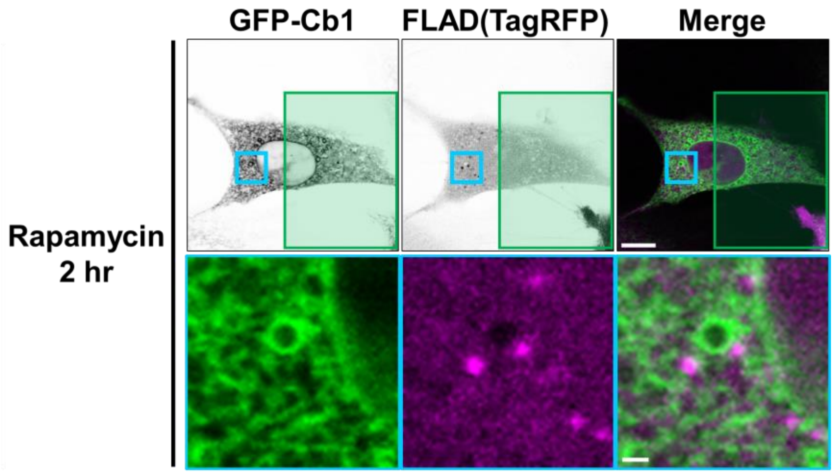

B

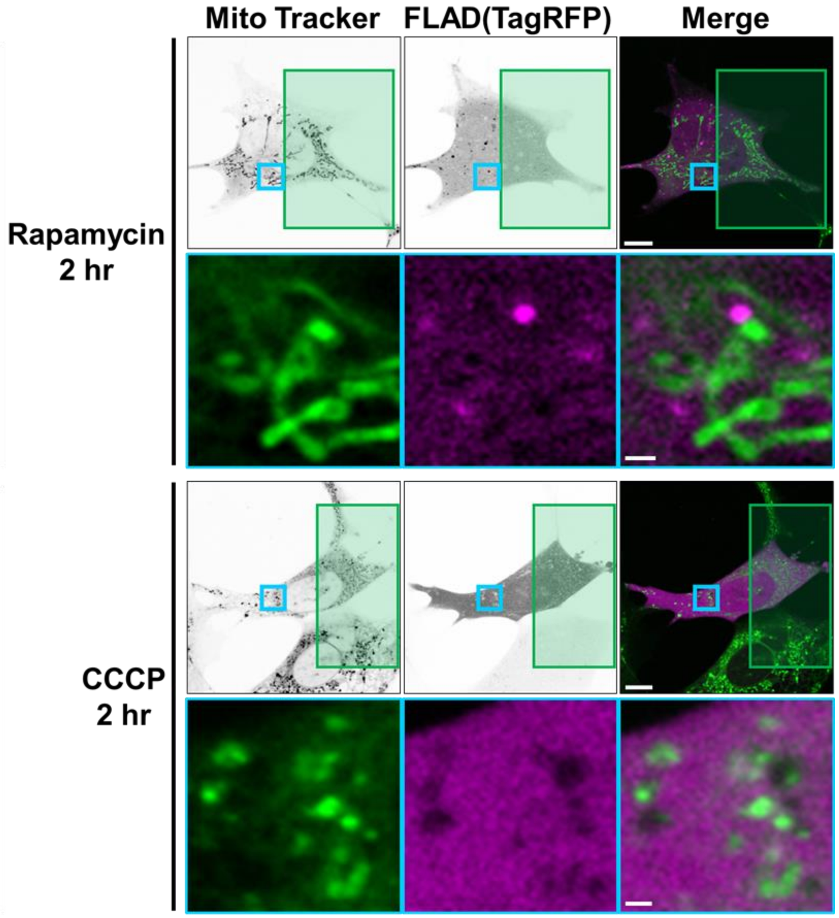

Supplemental Figure 6

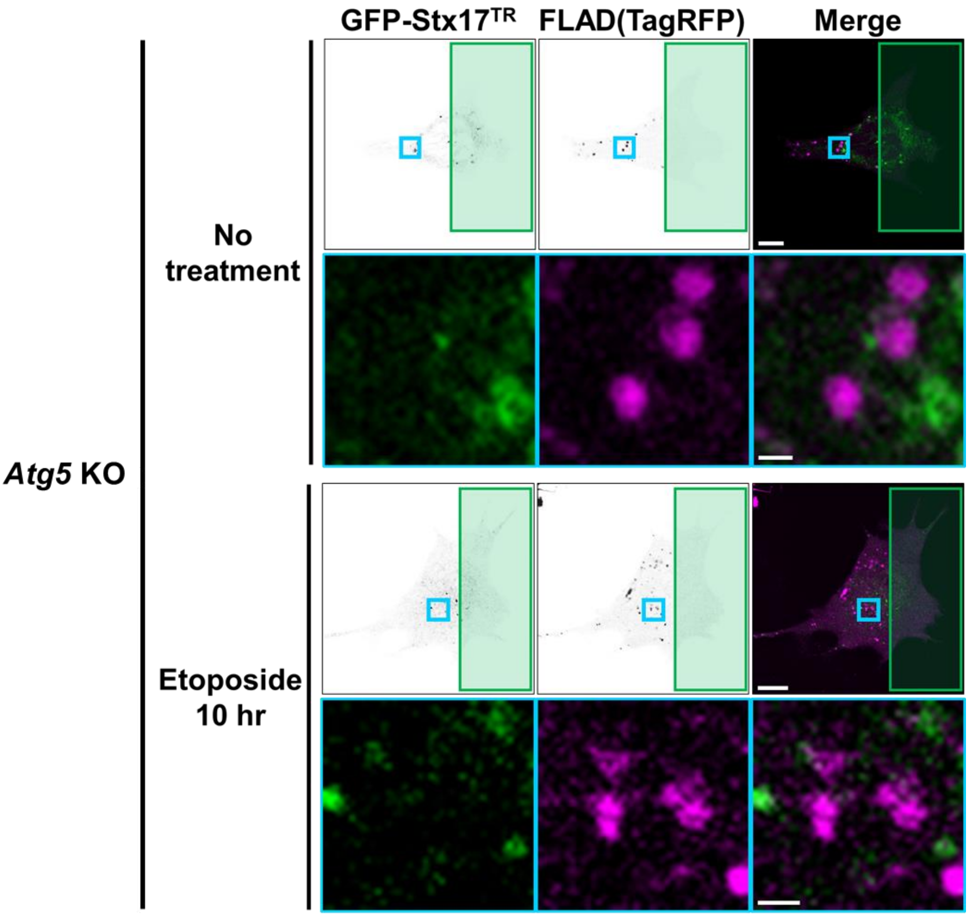

Supplemental Figure 7

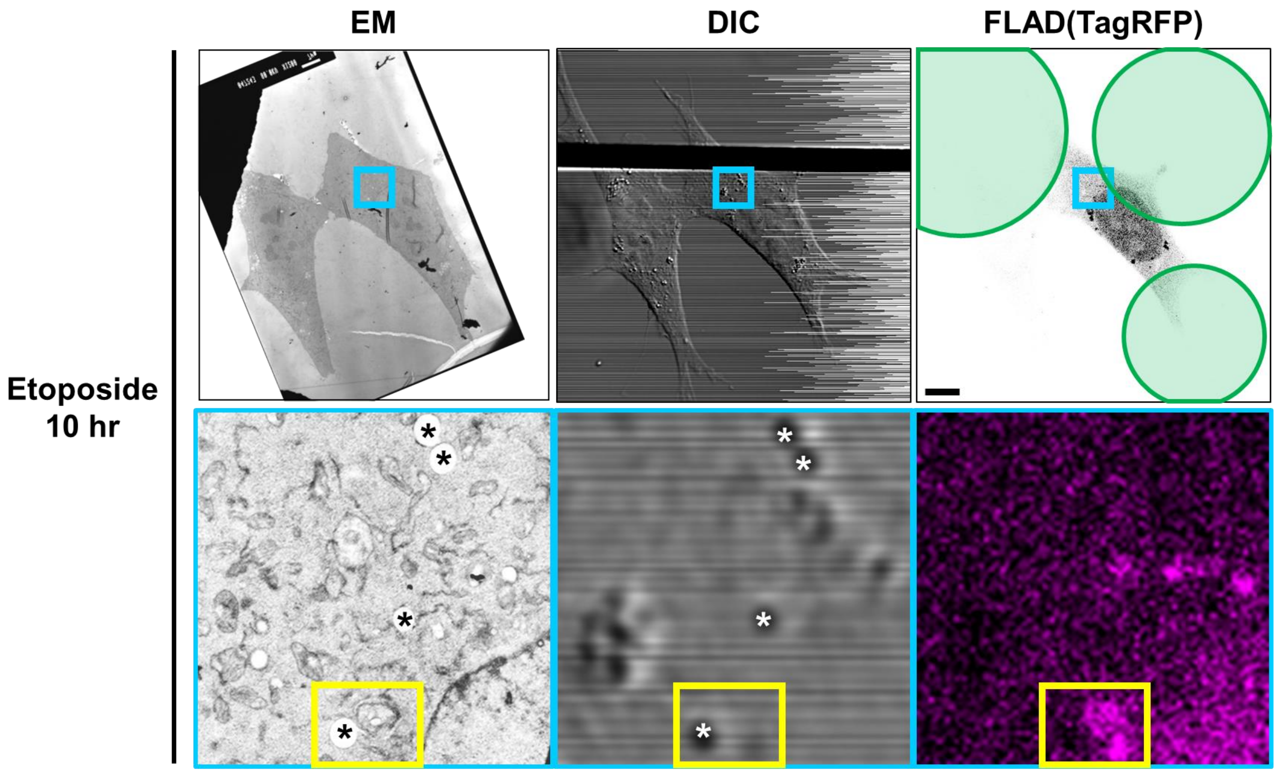

Supplemental Figure 8

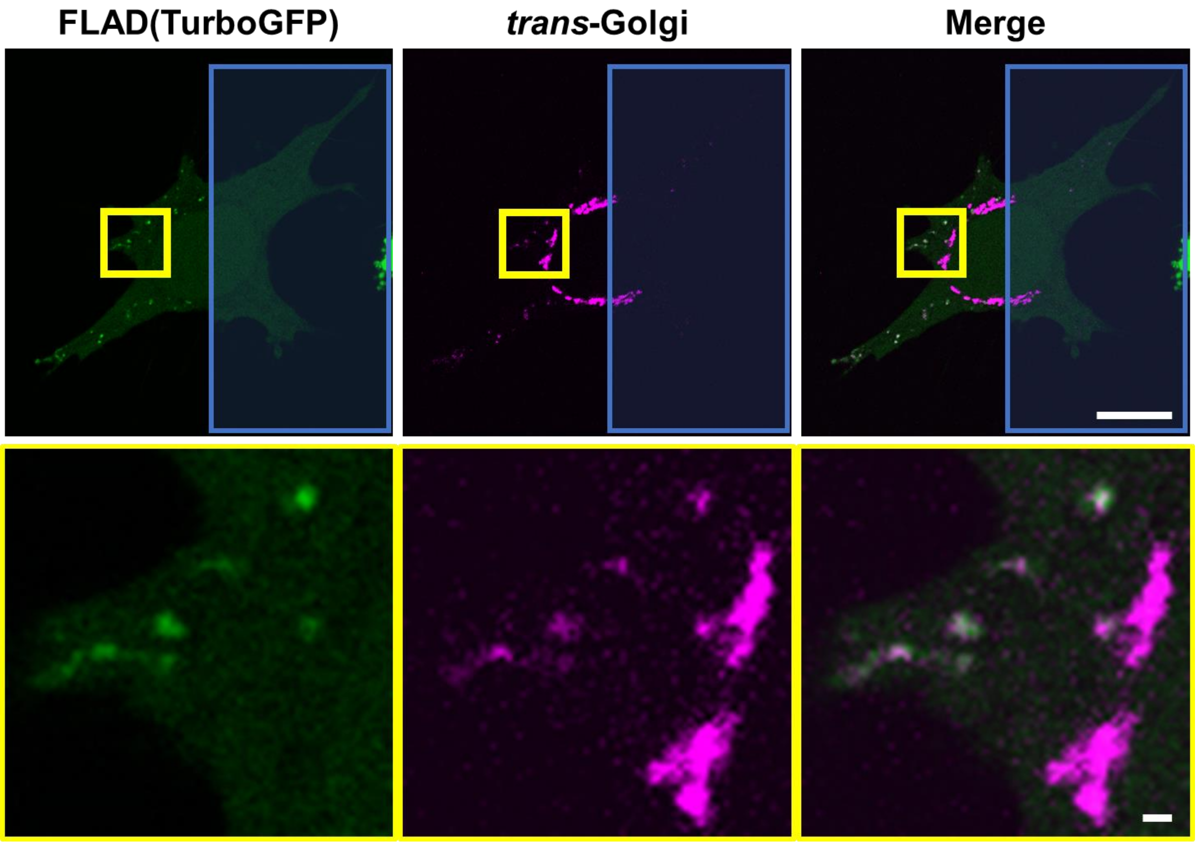

Supplemental Figure 9

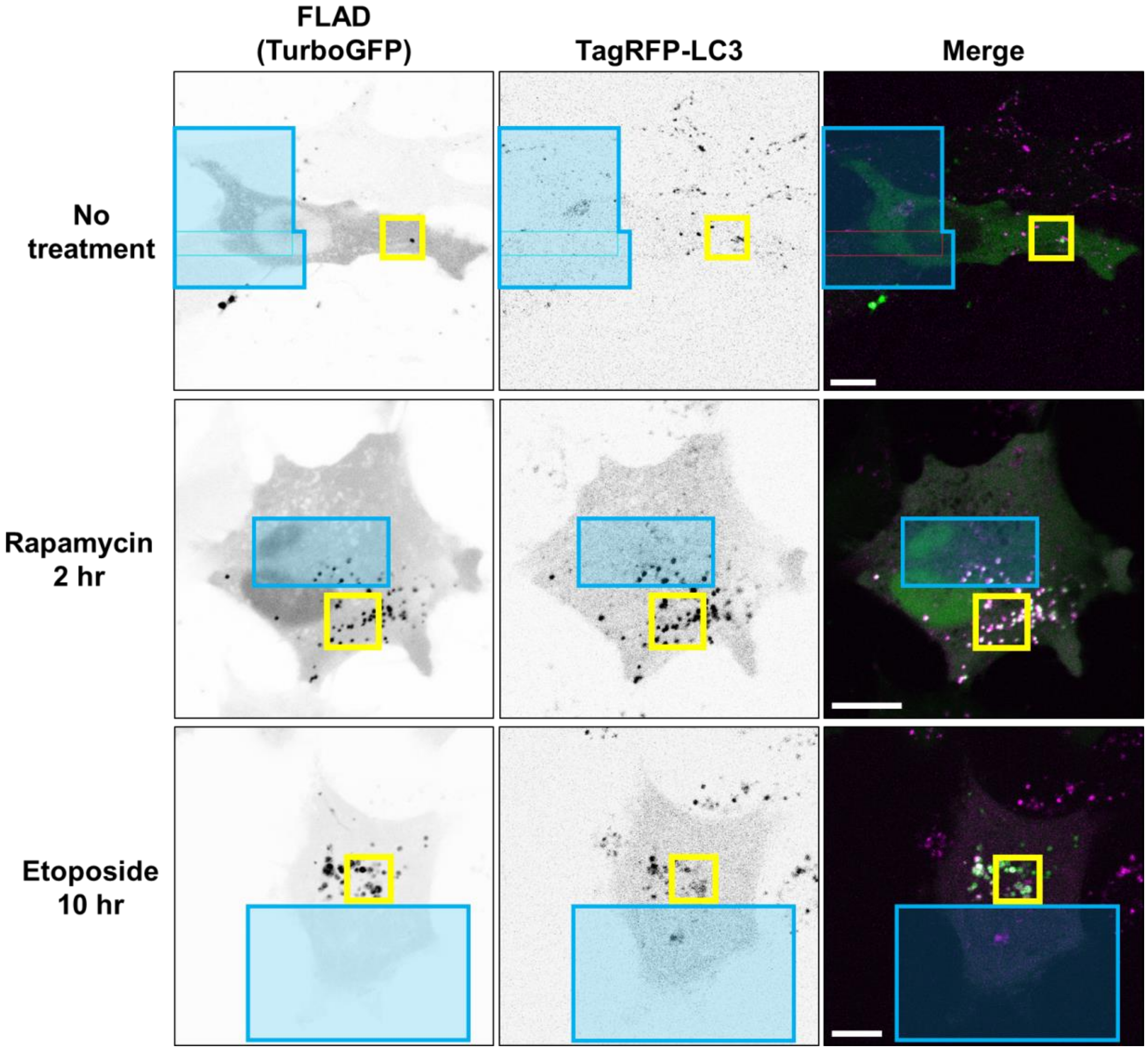

Supplement: Supplementary file 2 — Supplementary Information 2. [file 41598_2022_26430_MOESM2_ESM.pdf]
